# Diabetes, obesity, and recommended fruit and vegetable consumption in relation to food environment sub-types: a cross-sectional analysis of Behavioral Risk Factor Surveillance System, United States Census, and food establishment data

**DOI:** 10.1186/s12889-015-1819-x

**Published:** 2015-05-14

**Authors:** Cara L Frankenfeld, Timothy F Leslie, Matthew A Makara

**Affiliations:** Department of Global and Community Health, George Mason University, Fairfax, VA 22030 USA; Department of Geography and Geoinformation Science, George Mason University, Fairfax, VA 22030 USA

**Keywords:** Food environment, Diabetes, Obesity, Diet

## Abstract

**Background:**

Social and spatial factors are an important part of individual and community health. The objectives were to identify food establishment sub-types and evaluate prevalence of diabetes, obesity, and recommended fruit and vegetable consumption in relation to these sub-types in the Washington DC metropolitan area.

**Methods:**

A cross-sectional study design was used. A measure of retail food environment was calculated as the ratio of number of sources of unhealthier food options (fast food, convenience stores, and pharmacies) to healthier food options (grocery stores and specialty food stores). Two categories were created: ≤1.0 (healthier options) and >1.0 (unhealthier options). *k-*means clustering was used to identify clusters based on proportions of grocery stores, restaurants, specialty food, fast food, convenience stores, and pharmacies. Prevalence data for county-level diabetes, obesity, and consumption of five or more fruits or vegetables per day (FV5) was obtained from the Behavioral Risk Factor Surveillance System. Multiple imputation was used to predict block-group level health outcomes with US Census demographic and economic variables as the inputs.

**Results:**

The healthier options category clustered into three sub-types: 1) specialty food, 2) grocery stores, and 3) restaurants. The unhealthier options category clustered into two sub-types: 1) convenience stores, and 2) restaurants and fast food. Within the healthier options category, diabetes prevalence in the sub-types with high restaurants (5.9 %, *p* = 0.002) and high specialty food (6.1 %, *p* = 0.002) was lower than the grocery stores sub-type (7.1 %). The high restaurants sub-type compared to the high grocery stores sub-type had significantly lower obesity prevalence (28.6 % vs. 31.2 %, *p* <0.001) and higher FV5 prevalence (25.2 % vs. 23.1 %, *p* <0.001). Within the larger unhealthier options category, there were no significant differences in diabetes, obesity, or higher FV5 prevalence across the two sub-types. However, restaurants (including fast food) sub-type was significantly associated with lower diabetes and obesity, and higher FV prevalence compared to grocery store sub-type.

**Conclusions:**

These results suggest that there are sub-types within larger categories of food environments that are differentially associated with adverse health outcomes. These observations support the specific food establishment composition of an area may be an important component of the food establishment-health relationship.

## Background

Chronic health concerns, which include non-communicable diseases, are a serious nationwide and global health burden, and are projected to worsen in the coming years [1]. In the United States, more than 13 % of the population has three or more chronic diseases, and chronic diseases account for approximately seven out of ten deaths in Americans each year [2, 3]. Obesity is a well-established risk factor for heart disease, diabetes, stroke, several cancers, and other chronic diseases [4–6]. According to the International Agency for Research on Cancer, obesity is estimated to cause 14 % of cancer deaths in men and 20 % of cancer deaths in women [7]. An estimated 9.1 % of medical expenditures in the US are obesity-related, with a low estimate of $26.8 billion obesity-attributable medical expenditures per annum, [8] for a low estimate of $51.5 billion in medical expenditures per annum. The overwhelming health consequences and costs associated with obesity underscore the importance of identifying risk factors that can be modified through public health or policy interventions.

Nutrition and diet quality are key elements in the acquisition, control, and potential treatment of many chronic diseases and adverse health conditions. Higher consumption of fruits and vegetables has been associated with a lower risk of several diseases, including heart disease and several cancers [9–12]. The local food environment may influence individual (including food choices) and community health [13–15]. While individual food choices may be price-sensitive, community food choices are more heavily influenced by local availability [16]. Ideal food environments have available, accessible, affordable, and suitable food [17], which are factors that can associated with the socioeconomics of the area. Areas of comparatively higher socioeconomic status (SES) tend to have greater access to grocery stores [18], which may be considered a component of more ideal food environments. Residents of these areas have been observed to have lower BMI and a lower likelihood of obesity [18–20]. Conversely, areas of lower SES and higher material deprivation often lack grocery stores and instead are serviced by convenience stores and fast food establishments. Observations from other studies that suggest that neighborhood food options affect health outcomes [21, 22].

Higher obesity prevalence has been observed in residents of these areas, and the food environment may be a contributing factor [23]. The Washington DC Metropolitan area has approximately 5.6 million people [24]. In the District of Columbia itself, approximately 8 % of residents have been diagnosed with diabetes mellitus and 22 % are obese, underscoring the public health importance of chronic conditions [25]. There is considerable geographic disparity in the prevalence of these conditions with the residents in Wards 7 and 8 (southeastern DC) having prevalence of obesity at 35 % or higher, and residents of Wards 1 and 2 (central DC) having prevalence of obesity of 12 % or lower, with a similar pattern for having physician-diagnosed diabetes [26].

We previously published work evaluating spatial components to the retail food environment (RFE) of the Washington DC Metropolitan area, and observed that while grocery stores were consistently represented across different food environments, restaurants and fast food establishments had much greater variation [27], suggesting unique sub-types that make up a RFE. One objective of this study was to extend this prior work and evaluate characteristics of different sub-types of food environments within a RFE category (i.e., overall healthier options vs. unhealthier options environments). Another objective of this study was to quantify the association between RFE sub-types and prevalence of diabetes, obesity, and recommended fruit and vegetable consumption (5+ servings per day), at the block group level, while accounting for the SES of the block groups, in the Washington DC Metropolitan area.

## Methods

A cross-sectional analysis of RFE and health outcomes (diabetes and obesity prevalence) and behavior (recommended fruit and vegetable intake) was conducted. The overall goal of the analysis was to evaluate relationships with the RFE at the block-group level using imputed health outcome/behavior data. Food environment data was geocoded to the specific location of the food establishments, and thus could be aggregated to block-group level. Health behavior or outcome prevalence was available at the county level, and data was obtained from the Centers for Disease Control and Prevention (CDC) Behavioral Risk Factor Surveillance System (BRFSS). A multiple imputation model was used to generate imputed block-group level data based on US Census demographic and SES characteristic data of the block group. All data for this study are from publicly available, de-identified sources, and our IRB classified it as not human subjects research and did not require further review. Details about this overall process and analysis are provided in this section.

Food environment data were obtained from Dun & Bradstreet’s *Hoovers* listing (updated to April 2011) [28]. The *Hoovers* listing contains information on the location (on an X,Y level based on their geocoding) and NAICS (North American Industrial Classification System) sector of each establishment within predefined food environment categories [29]. The number and types of food establishments were enumerated for the District of Columbia, Maryland, and Virginia parts of the DC Metropolitan area, as defined by the United States Office of Management and Budget. There were 13,326 establishments across six sectors: pharmacies and drug stores (NAICS 44611, *n* = 847), restaurants and other eating places (NAICS 722511, *n* = 3898), supermarkets and other grocery (except convenience) stores (NAICS 44511, *n* = 2065), specialty food stores (NAICS 4452, *n* = 1042), convenience stores (NAICS 44512, *n* = 2178), and fast food restaurants (NAICS 722513, *n* = 3296). The distinction between restaurants and fast food is determined by whether there is waiter/waitress service to patrons, and establishments such as coffee shops are classified as fast food. Specialty food stores includes meat, fish, and seafood, and fruit and vegetable markets; baked goods; and, confectionery and nut stores. A RFE is the collection of retail businesses available in communities as options for purchasing food. RFE calculation was informed by published methods, and applied to the predefined establishment types available from the *Hoovers* listing [30, 31]. Differing from the CDC’s percentage-based method, we calculated the RFE as a ratio of counts of more unhealthier food options to sources of more healthier food options: (fast food + convenience stores + pharmacies + restaurants)/(grocery stores + specialty food stores). The block-group aggregation process included a half-mile buffer to account for boundary effects of the block group to account for boundary effects.

Prevalence of diabetes and obesity, estimated by the Behavioral Risk Factor Surveillance System (BRFSS), was obtained from the CDC for 2010 [32]. BRFSS is a cross-sectional telephone survey conducted by state health departments, and is used to collect prevalence data for risk behaviors and preventive health practices among adult residents in the United States. The survey includes landline and cellular telephone samples. A disproportionate stratified sampling procedure is used to draw landline telephone numbers from strata based on the presumed density of telephone household numbers. The cellular telephone sample is randomly generated from a sampling frame of confirmed cellular area code and prefix combinations. Approximately 20 % of interviews are completed with cellular phones. Data from out-of-state interviews (i.e., individuals who have moved into other states) are transferred to the appropriate states. The following specific BRFSS questions were used to evaluate diabetes and obesity: “Have you ever been told by a doctor that you have diabetes?”; “About how much do you weigh without shoes?”; and, “About how tall are you without shoes?” The analysis focused on individuals with prevalent diabetes. Individuals who reported diagnosis of prediabetes or diabetes during pregnancy were not included as having prevalent diabetes. Obesity was defined as BMI ≥30 kg/m^2^.

Fruit and vegetable consumption for each area was also obtained for 2009. Fruit and vegetable consumption data was not collected in 2010. On the BRFSS questionnaire, participants are asked to respond numerically to six questions about intake of fruits and vegetables (“How often do you drink fruit juices, such as orange, grapefruit, or tomato?”; “Not counting juice, how often do you eat fruit?”; “How often do you eat green salad?”; “How often do you eat potatoes not including French fries, fried potatoes, or potato chips?”; “How often do you eat carrots?”; and, “Not counting carrots, potatoes, or salad, how many servings of vegetables do you usually eat?”). Participant responses are used to calculate frequency per day and categorize individuals into groups of consuming 5+ or <5 fruit or vegetable consumption per day, and this dichotomized variable is part of the calculated variables provided by BRFSS. A general dietary guideline is for individuals to consume five or more servings of fruits or vegetables per day [33].

Census data from 2010 was used to characterize demographic and SES attributes of geographic regions. Demographic and SES variables used reflect overall characteristics, socioeconomic inequality, or deprivation in geographic areas [34, 35]. These variables included the percentage of: males, individuals age 18 years and older, individuals age 65 years and older, households with female head of household and children present, households with no vehicle present, workforce employed in management, individuals using public assistance, families in poverty, individuals of White race, individuals of Black race, and individuals of Hispanic ethnicity. Median income was also included. Data were extracted from American Factfinder2 at the county and block group levels [36]. Block groups are statistical divisions of census tracts and typically include between 600 and 3000 residents. A block group consists of clusters of blocks within the same census tract and usually covers a contiguous area. Block group boundaries do not cross state, county, or census tract boundaries. Block groups with no Census demographic data (n = 549), indicating no or few residents, or incomplete demographic data for the variables needed for the multiple imputation model (n = 82), were excluded from the analysis. These demographic and SES characteristics explained 65 %, 44 %, and 67 % of the variability in diabetes, obesity, and FV5, respectively, based on the linear regression R-squared values.

A multiple imputation model was used to predict the block-group level prevalence of diabetes, obesity, and FV5. Metropolitan/micropolitan data for counties in the United States that did not include the counties in the analysis (i.e., the DC Metropolitan area) was obtained from the Selected Metropolitan/Micropolitan Area Risk Trends of BRFSS (SMART BRFSS) [32]. Data across the US provided a varied distribution of health condition prevalence, which provided more stability in the models at more extreme values. Demographic and SES characteristics (listed in prior paragraph) for these areas were used as the predictors (x-variables) in a multivariable normal regression imputation model to predict block-group health behavior/outcome prevalence. The metropolitan/micropolitan-level data (excluding the DC metropolitan area) was considered as complete data and the block-group DC metropolitan area was considered as being missing data. This method controlled for the demographic and SES variables in the prediction of diabetes, obesity, and FV5 prevalence. An iterative Markov chain Monte Carlo (MCMC) method was used [37]. The multiple imputation was performed 10 times to generate 10 different datasets with imputed prevalence. All of the analyses evaluating the health outcome/behavior prevalence utilized the appropriate estimation procedures in order to obtain valid estimates of variability (e.g., standard errors of means).

The RFE ratio was divided into two categories, which are referred to here as “healthier options” and “unhealthier options”. The division was ≤1.0, which indicates environments with higher number of sources of more healthy foods than sources of more unhealthy foods, and >1.0, indicating the converse. Restaurants may be neutral in terms of healthier vs. unhealthier, and were not included in the initial calculations of this division. To evaluate sub-types of food establishments that create healthier and unhealthier options areas, statistical clustering was used. Clustering was based on the proportion of food establishments because the number of establishments will drive the clusters (i.e., most of the areas with overall high number of establishments will be in clusters together, regardless of the composition of the area), and proportion does provide information about food establishment options available to people in a geographic area. *k-*means is a non-hierarchical method of clustering observations into groups, [38] and it was used to identify two or three clusters within each RFE category based on densities of grocery stores, restaurants, specialty food, fast food, convenience stores, and pharmacies. The “cluster kmeans” command in Stata was used to generate clusters. The similarity/dissimilarity measured used was Euclidean distance. The *k-*means clustering was performed 10 times using initially randomly generated seed numbers that were consistently used for replicability. For the healthier options category, three clusters (sub-types) consistently emerged based on the high percentage of 1) grocery stores; 2) restaurants; and, 3) specialty food, and two clusters emerged for the unhealthier options category based on the high percentage of 1) restaurants, and 2) convenience stores. During each of the 10 clustering runs, each block group was assigned to one of the clusters, which could vary based on the seed number for the run. The most frequently assigned cluster (mode) for the block group was assigned for statistical analysis of health outcome/behavior and food environment sub-type.

Means and standard deviations of proportions were calculated to describe the demographic and SES characteristics within the healthier and unhealthier option categories, and t-tests were used to evaluate for differences across the categories. Means and standard deviations were also calculated to describe the percentage of food establishment types in each sub-type (cluster) within the healthier and unhealthier options categories. Statistical comparisons were not performed for food establishment differences because an objective of clustering is to make the categories as dissimilar as possible, and the statistical significance of this dissimilarity is not meaningful to this analysis. Linear regression was used to evaluate block-group level prevalence of diabetes, obesity, and FV5 in relation to the healthier and unhealthier options, and sub-types within and across those options categories. For the healthier option category, grocery store sub-type served as the reference group. For the unhealthier option category, restaurant sub-type served as the reference group. Comparison of health outcomes/behavior was conducted across all of the sub-types using the unhealthier options restaurant sub-type (largest category and lowest prevalence of diabetes and obesity) as the reference group. Analyses were performed using Stata 11.0 (Stata Corp, College Station, TX). A map for the food environment sub-types was created using ArcGIS.

## Results

In the aggregate metropolitan area, the mean RFE ratio was 2.06 (95 % CI: 2.01, 2.10). The ratio was significantly lower (more favorable towards higher proportion of healthier options) within the District of Columbia (mean = 1.56, 95 % CI: 1.49, 1.63, *p* <0.001) compared to Maryland (mean = 2.01, 95 % CI: 1.93, 2.09) and Virginia (mean = 2.17, 95 % CI; 209, 2.24, *p* <0.001). Overall, the percentage of block groups that were in the healthier options category (RFE ≤1.0) was similar across the aggregate areas of DC (25 %), Maryland (27 %), and Virginia (21 %).

Overall food environment distributions and SES characteristics by categories of healthier and unhealthier options are presented in Table 1. As expected given the RFE ratio calculation, grocery market and specialty food densities were higher in the healthier options category, and fast food, restaurant, convenience store, and pharmacy densities were higher in the unhealthier options category. Variation in demographics and SES across categories was observed for gender, younger age, household characteristics, Hispanic ethnicity, and income. There were no differences in proportions of older age, Black race, and employment.

**Table 1:** Food establishment and SES characteristics across retail food environment (RFE) categories

Block group characteristics	Healthier options (RFE ratio ≤1.0)	Unhealthier options (RFE ratio >1.0)
Block groups (n)	769	2458
Supermarket^1^ (%)	21.2 ± 17.0 (0–75)	12.8 ± 7.9 (0–40)***
Restaurant (%)	16.9 ± 17.9 (0–77)	23.3 ± 12.7 (0–67)***
Specialty food (%)	14. 7 ± 16.7 (0–75)	6.0 ± 6.0 (0–38)***
Fast food (%)	7.1 ± 9.8 (0–40)	24.2 ± 11.4 (0–67)***
Convenience store (%)	8.8 ± 11.1 (0–40)	18.7 ± 11.8 (0–86)***
Pharmacy (%)	2.5 ± 5.8 (0–40)	6.8 ± 7.4 (0–75)***
Male (%)	49.0 ± 2.7 (31–70)	48.8 ± 3.3 (29–90)*
Age 18 years or older (%)	74.4 ± 6.1 (47–100)	76.5 ± 6.6 (51–100)***
Age 65 years or older (%)	10.7 ± 8.1 (0–83)	10.8 ± 8.1 (0–94)
Female head of household with children (%)	50.6 ± 14.5 (0–100)	50.0 ± 13.8 (0–100)
Rented housing (%)	21.7 ± 23.3 (0–100)	31.8 ± 27.4 (1–100)***
No vehicle in household (%)	2.4 ± 4.4 (0–45)	3.7 ± 4.0 (0–49)***
Employed in management (%)	49.5 ± 15.0 (3–82)	50.0 ± 16.4 (3–93)
Using public assistance (%)	3.1 ± 3.5 (0–23)	3.7 ± 4.0 (0–44)**
Family poverty (%)	3.6 ± 4.2 (0–27)	4.3 ± 5.2 (0–100)**
White race (%)	66.3 ± 26.9 (1–100)	62.3 ± 25.3 (0–99)***
Black race (%)	20.2 ± 25.2 (0–95)	21.3 ± 23.6 (0–98)
Hispanic ethnicity (%)	9.6 ± 12.7 (0–94)	12.5 ± 12.5 (0–90)***
Income (median per 10,000 dollars)	5.4 ± 1.8 (2–12)	5.3 ± 1.8 (0–15)*

Mean ± standard deviation and (range) of block group percentages are presented unless otherwise noted

^1^Percentage of food establishments is calculated as number of food establishment (e.g., grocery stores)/total count of food establishments (supermarkets, restaurants, specialty food, fast food, convenience stores, and pharmacies)

*p <0.05, **p <0.01, ***p <0.001 for mean values compared to healthy block groups, using a two-tailed *t*-test

Table 2 displays the food environment sub-types within the healthier and unhealthier options categories. The healthier options category clustered into three sub-types associated with higher percentages of: 1) specialty food, 2) grocery stores, and 3) restaurants. The grocery stores and restaurants sub-types also had higher percentages of fast food and convenience stores than the specialty food sub-type. The unhealthier options category clustered into two sub-types associated with higher percentages of: 1) convenience stores, and 2) restaurants and fast food. Grocery stores, specialty food, fast food, and pharmacy percentages were similar across these two sub-types in the unhealthier options category.

**Table 2:** Mean ± standard deviation of block group food establishment types percentages^1^ in retail food environment (RFE) category sub-types

Food environment categories	Block groups (n)	Markets	Restaurants	Specialty food	Fast food	Convenience	Pharmacies
Healthier options (RFE ratio ≤1)							
Sub-type 1: Grocery stores	376	28.1 ± 18.4	8.2 ± 9.6	18.3 ± 20.1	7.3 ± 10.2	12.3 ± 12.4	2.8 ± 6.5
Sub-type 2: Restaurants	213	20.0 ± 9.4	35.2 ± 10.8	11.9 ± 10.4	10.7 ± 9.5	6.0 ± 7.0	3.0 ± 4.8
Sub-type 3: Specialty Food	180	8.3 ± 12.5	13.5 ± 21.9	10.6 ± 13.1	2.4 ± 7.0	4.8 ± 9.7	1.4 ± 5.3
Unhealthier Options (RFE ratio >1)							
Sub-type 1: Restaurants and fast food	1520	12.5 ± 6.8	29.1 ± 10.6	5.9 ± 5.2	27.6 ± 9.8	12.6 ± 6.2	6.5 ± 6.3
Sub-type 2: Convenience stores	938	13.3 ± 9.4	13.8 ± 9.7	6.2 ± 7.1	18.8 ± 11.7	29.3 ± 11.5	7.2 ± 8.9

^1^Percentage of food establishments is calculated as number of food establishment (e.g., grocery stores)/total count of food establishments (supermarkets, restaurants, specialty food, fast food, convenience stores, and pharmacies) x 100

There were significant differences in the prevalence of diabetes, obesity, and FV5 across the sub-types within the healthier options category and across all sub-types (Table 3). Within the broad healthier options category, the sub-types with higher restaurants and higher specialty food had lower diabetes prevalence than the grocery stores sub-type. The restaurants sub-type also had lower obesity prevalence and higher FV5 prevalence than the grocery stores sub-type. Within the broad unhealthier options category, there were no differences across the sub-types. Across both categories and using grocery stores sub-type as the reference, diabetes prevalence was significantly lower in all of the other sub-types, and obesity and FV5 were more favorable in some of the other sub-types.

**Table 3:** Block-group level prevalence (95 % confidence interval) of predicted diabetes, obesity, and FV5 in retail food environment (RFE) categories^

Food environment categories	Block groups (n)	Diabetes	Obesity	5+/day Fruit and Vegetable Intake
Healthier Options (RFE ratio ≤1)				
Sub-type 1: Grocery Stores	376	7.1 (6.3, 7.9)	31.2 (30.6, 31.9)	23.1 (21.7, 24.5)
Sub-type 2: Restaurants	213	5.9 (5.0, 6.8)**^,††^	28.6 (27.8, 29.4)**^,††^	25.2 (23.8, 26.5)**^,††^
Sub-type 3: Specialty Food	180	6.1 (5.0, 7.2)**^,††^	31.1 (30.1, 32.1)	23.6 (22.3, 25.0)^†^
Unhealthier Options (RFE ratio >1)				
Sub-type 1: Restaurants and fast food	1520	6.0 (4.9, 7.1)^††^	29.6 (28.5, 30.7)^††^	24.4 (23.1, 25.9)^††^
Sub-type 2: Convenience Stores	938	6.1 (4.9, 7.3)^††^	30.9 (29.5, 32.4)	23.7 (22.1, 25.3)

^Diabetes, obesity, and FV5 predicted from multiple imputation using variables in Table 1

*p <0.05, **p <0.01, ***p <0.001 for comparison of diabetes, obesity, or fruit and vegetable intake prevalence compared to the reference group within the healthier options (reference: grocery stores) and unhealthier options (reference: restaurants and fast food)

^†^ *p* <0.05, ^††^ *p* <0.01, ^†††^ *p* <0.001 for comparison of diabetes, obesity, or fruit and vegetable intake prevalence compared to the reference group of “Healthier Options Sub-Type 1: Grocery Stores” across healthier and unhealthier options categories (in the entire group)

## Discussion

One objective of this study was to extend this prior work and evaluate characteristics of different sub-types of food environments within a RFE category (i.e., overall healthier options vs. unhealthier options environments). These results suggest that food environments, at the block-group level, that are classified as having a higher proportion of healthier options clusters into sub-types based on grocery stores, restaurants, and specialty food. However, specialty food stores were not a dominating driver of the third sub-type, and similar proportion of specialty food and restaurants were observed. Food environments that were classified as having a lower proportion of healthier options cluster into two sub-types, one dominated by convenience stores and the other by restaurants and fast food establishments.

Another objective of this study was to quantify the association between RFE sub-types and prevalence of diabetes, obesity, and recommended fruit and vegetable consumption (5+ servings per day), at the block group level, while accounting for the SES of the block groups, in the Washington DC Metropolitan area. Arguably, the most interesting observation is that, within a food environment classified as “healthier options” based on an overall ratio of sources of unhealthier food to sources of healthier food, there are distinct sub-types that give rise to the overall food environments, and these sub-types are differentially associated with health outcomes. The particular food environment sub-types associated with better health outcomes/behaviors were characterized by higher percentage of restaurants or specialty food, compared with the grocery market sub-type. Within the overall unhealthier food environments, there were no differences across the sub-types. Additionally, the healthier options grocery market sub-type and unhealthier options convenience store sub-type had similar prevalence of obesity and FV5, supporting that the specific food establishment composition of an area may be an important component of the food establishment-health relationship. A possible associated factor is that areas with grocery stores may be in areas that are more reliant on car traffic and are less pedestrian friendly. Such neighborhoods may be associated with other variables, such as exercise patterns, that also influence diabetes and obesity.

Lower SES is a risk factor for diabetes and obesity [39–41]. Areas of comparatively higher SES tend to have greater access to grocery stores, and residents of these areas have shown significant associations with decreased BMI and lower odds of obesity. Similarly, areas of lower SES and higher material deprivation lack proximity to grocery stores and food choices are often limited to convenience stores and fast food establishments. Higher obesity prevalence has been observed in residents of these areas. However, SES is composed of numerous factors that are also risk factors for adverse health conditions, and less is known about the food environment independent of neighborhood SES characteristics. The design of our analysis controlled for numerous potential confounding factors, including neighborhood demographic and SES characteristics, suggesting that association between food environment and health may not be limited to economically deprived areas, and future work should evaluate the role of the food environment on adverse health outcomes across varied demographic and SES areas at different levels of geography.

The Washington DC Metropolitan region includes the densely populated urban area of the District of Columbia and some parts of the suburban and rural areas in the surrounding states of Virginia, Maryland, and West Virginia. RFE for this study was measured by the ratio of more unhealthier food options (fast food, restaurants, convenience stores, and pharmacies) to more healthier food options (grocery stores and specialty food stores). There are different measures that have been used to evaluate the food environment based on counts or densities of food establishments, such as the Retail Food Environment Index (RFEI) used by the California Center for Public Health Advocacy (CCPHA), which is a ratio of (fast food restaurants and convenience stores) to (supermarkets, other grocery stores, and produce vendors) [42]. The CDC also uses a modified RFEI that is a percentage-based measure of the number of health food retailers divided by the total number of healthy and less healthy food retailers [43]. There is no gold standard measure of the RFE. The measure we used here was more similar to the CCPHA, but analyses also included restaurants in the overall count of food establishments (denominator for proportion calculations) in order to not exclude a substantial portion of the food establishments in the DC Metropolitan Area. The mean RFE ratio for the District of Columbia metropolitan region suggests there are twice as many unhealthier food options than healthier food options. However, there was a wide variation in the RFE ratio within the metropolitan area. In other work, we observed that this distribution was not solely a result of a higher frequency of grocery stores, which suggested that changing the grocery store availability may not be the most effective route to favorably altering the food environment [27].

The differences in health behavior and outcome prevalence across sub-types are notable in healthier options and across healthier options and unhealthier options categories, suggesting that some healthier options environments are associated with poorer health outcomes. If these observations contribute to further work in the field that identifies ways to mitigate health outcomes through the food environment across all levels of the food environment, this could have tremendous public health impact. For example, higher diabetes prevalence can add considerably to health care costs. The American Diabetes Association reported that expenditures attributable to diabetes were greater than $245 billion per year across the population and annual per capita health care costs with $176 billion as the estimated direct costs [44].

There are some notes of caution in the interpretation of these results. The study was cross-sectional and directionality of the association cannot be established with this design. As such, it is not possible to evaluate within this study whether unhealthier RFE are causes of adverse health outcomes or behaviors. The temporality and timing of food environment influence will be an another important consideration for policies or understanding etiology and future studies at the individual level and conducted longitudinally can provide complementary information to our observations. Temporality may also be an issue due the differences in years of the BRFSS data and food establishment data. The food establishment data is updated continuously. There will have been some changes in the food establishments after the BRFSS data collection, and this is a noted limitation of the analysis. However, the majority of the food establishments will be reflective of what was present at the time of BRFSS data collection. Demographic and socioeconomic variables were inherently controlled for the health outcomes prevalence by the imputation method, but residual confounding and confounding by unmeasured characteristics is possible. For example, unmeasured characteristics could include whether certain population groups that cluster geographically have particular beliefs around certain foods or types of food. The ecological design is appropriate for this specific analysis because it is a way to evaluate the RFE on the aggregate level adverse health outcomes. This can be relevant to evaluating whether food environment changes at the aggregate level (i.e., policies or infrastructure) influence health. However, the ecological design does not allow for evaluating whether macro-level RFE effects influence individual-level behaviors or outcomes. Further work using individual-level observational data will inform etiologic mechanisms by which the RFE affects health. Studies that can utilize individual-level geocoded health data are needed to address the questions about etiologic mechanisms. This analysis allows for study of population-level health in relation to food environment proximity, but it is not possible to study utilization of a given food environment. The geographic focus of this study provides valuable information to the District of Columbia metropolitan area, but may not be generalizable to other regions. The U.S. Census and BRFSS data are national; thus, methodology used in this analysis could be used for future studies within other areas or across the U.S.

## Conclusions

Overall, the results of this study suggest that the immediate food environment is significantly associated with prevalence of diabetes, obesity, and 5+ fruit or vegetable consumption across the DC Metropolitan area. These results are consistent with expectations of the association between food environment and health conditions. However, a notable observation in this study was that within environments with higher density of healthier food sources compared to density of unhealthier food sources, there are different types of sub-types. These sub-types are differentially associated with diabetes, obesity, and 5+ fruit or vegetable consumption. Specifically, areas with a relatively higher density of grocery stores had higher prevalence of diabetes and obesity, and lower prevalence of fruit or vegetable consumption, compared to the other sub-environments with a particular level of RFE. These relationships have not been widely studied, and our results contribute to a foundation for future work to evaluate the role of particular distributions of food establishments within areas that have an overall favorable ratio of sources of healthier food to sources of unhealthier food.

Evaluation of the immediate food environment of an area may provide public health professionals and policy makers with information regarding the most effective level of geography at which changes in the food environment could improve health (e.g., the effectiveness of mass campaigns vs. local level interventions). Information at the block-group level may help to inform local strategies, such as community outreach or activities, or feedback to higher level urban planning. Future studies could evaluate legislative districts or municipalities, which could be informative to policy makers. To extend beyond cross-sectional, ecological work, future studies of food-related environmental and individual-level factors will help elucidate targets for public health intervention to reduce adverse health conditions, through means such as modification of the food environment or individual-level interventions to make healthier choices within the food environment. It may be beneficial to extend this work to other metropolitan regions to assess if there is consistency across regions. The results of this study likely extend to other geographic areas, and this work provides a foundation to evaluate other areas.

## References

[1] Preventing chronic disease: a vital investment, [Accessed April 11, 2012. From: http://www.who.int/chp/chronic_disease_report/full_report.pdf]

[2] Paez KA, Zhao L, Hwang W (2009). Rising out-of-pocket spending for chronic conditions: a ten-year trend. Health Aff (Millwood).

[3] Kung HC, Hoyert DL, Xu J, Murphy S: Deaths. Final Data for 2005. In: National Vital Statistics Reports. Centers for Disease Control and Prevention; 2008. 18512336

[4] Eckel RH (1997). Obesity and Heart Disease: a statement for healthcare professionals from the Nutrition Committee, American Heart Association. Circulation.

[5] Diet, Nutrition, and the Prevention of Chronic Diseases: Report of a Joint WHO/FAO Consultation

[6] World Cancer Research Fund, American Institute for Cancer Research: policy and action for cancer prevention. Food, nutrition, and physical activity: a global perspective; 2009.

[7] International agency for research on cancer: weight control and physical activity: IARC Press; 2002.

[8] Finkelstein EA, Fiebelkorn IC, Wang G. National medical spendng attributable to overweight and obesity: how much, and who’s paying? Health Affects (Millwood) 2003, Suppl web Exclusives:W3-219-226. 10.1377/hlthaff.w3.219 14527256

[9] Bazzano LA, Serdula MK, Liu S (2003). Dietary intake of fruits and vegetables and risk of cardiovascular disease. Curr Atheroscler Rep.

[10] Gonzalez CA (2006). Nutrition and cancer: the current epidemiological evidence. Br J Nutr.

[11] Key TJ, Schatzkin A, Willett WC, Allen NE, Spencer EA, Travis RC (2004). Diet, nutrition and the prevention of cancer. Public Health Nutr.

[12] van't Veer P, Jansen MC, Klerk M, Kok FJ (2000). Fruits and vegetables in the prevention of cancer and cardiovascular disease. Public Health Nutr.

[13] Larson NI, Story MT, Nelson MC (2009). Neighborhood environments: disparities in access to healthy foods in the U.S.. Am J Prev Med.

[14] Whelan A, Wrigley N, Warm D, Cannings E (2002). Life in a food desert. Urban Stud.

[15] United States Department of Agriculture (USDA), Economic Research Service: access to affordable and nutritious food: measuring and understanding food deserts and their consequences; June 2009.

[16] Morland K, Diez Roux AV, Wing S (2006). Supermarkets, other food stores, and obesity: the atherosclerosis risk in communities study. Am J Prev Med.

[17] Wrigley N (2002). 'Food Deserts' in British Cities: policy context and research priorities. Urban Stud.

[18] Rose D, Hutchinson PL, Bodor JN, Swalm CM, Farley TA, Cohen DA (2009). Neighborhood food environments and Body Mass Index: the importance of in-store contents. Am J Prev Med.

[19] Bodor JN, Rice JC, Farley TA, Swalm CM, Rose D (2010). The association between obesity and urban food environments. J Urban Health.

[20] Michimi A, Wimberly MC (2010). Associations of supermarket accessibility with obesity and fruit and vegetable consumption in the conterminous United States. Int J Health Geogr.

[21] Apparicio P, Abdelmajid M, Riva M, Shearmur R. Comparing alternative approaches to measuring the geographical accessibility of urban health services: distance types and aggregation-error issues. Int J Health Geogr. 2008, 7(7). 10.1186/1476-072X-7-7 PMC2265683 18282284

[22] Smoyer-Tomic KE, Spence JC, Amrhein C (2006). Food deserts in the prairies? Supermarket accessibility and neighborhood need in Edmonton, Canada. Prof Geogr.

[23] Millstein RA, Yeh HC, Brancati FL, Batts-Turner M, Gary TL (2009). Food availability, neighborhood socioeconomic status, and dietary patterns among blacks with type 2 diabetes mellitus. Medscape J Med.

[24] Metropolitan and Micropolitan Statistical Areas Totals: Vintage 2011 [http://www.census.gov/popest/data/metro/totals/2011/index.html]

[25] BRFSS 2008 Annual Report [http://doh.dc.gov/sites/default/files/dc/sites/doh/publication/attachments/BRFSS_Annual_Report_2008_0.pdf]

[26] Annual Health Report Behavioral Risk Factor Surveillance System.

[27] Leslie TF, Frankenfeld CL, Makara MA (2012). The spatial food environment of the DC Metropolian area: clustering, co-location, and categorical differentiation. Appl Geogr.

[28] Dun and Bradstreet: Dun and Bradstreet Selectory Database; 2011.

[29] North American Industry Classification System [http://www.census.gov/eos/www/naics/]

[30] Jilcott S, McGuirt J, Imai S, Evenson K (2010). Measuring the retail food environment in rural and urban North Carolina counties. J Public Health Manag Pract.

[31] Koleilat M, Whaley SE, Afifi AA, Estrada L, Harrison GG. Understanding the relationship between the retail food environment index and early childhood obesity among WIC participants in Los Angeles County using GeoDa. Online J Public Health Inform. 2012, 4(1). 10.5210/ojphi.v4i1.3936 PMC3615800 23569623

[32] Office of Surveillance, Epidemiology, and Laboratory Services. Behavioral Risk Factor Surveillance System [http://www.cdc.gov/brfss/]

[33] Dietary Guidelines for Americans [http://www.health.gov/dietaryguidelines/]

[34] Messer LC, Laraia BA, Kaufman JS, Eyster J, Holzman C, Culhane J (2006). The development of a standardized neighborhood deprivation index. J Urban Health: Bull New York Acad Med.

[35] Carstairs V (1995). Deprivation Indices: their Interpretation and use in relation to health. J Epidemiol Community Health.

[36] American FactFinder [http://factfinder2.census.gov/main.html]

[37] Carpenter JR, Kenward MG (2013). Multiple imputation and its application.

[38] McCune B, Grace JB. Analysis of ecological communities. Gleneden Beach, OR: MjM Software Design; 2002.

[39] Agardh E, Allebeck P, Hallqvist J, Moradi T, Sidorchuk A (2011). Type 2 diabetes incidence and socio-economic position: a systematic review and meta-analysis. Int J Epidemiol.

[40] El-Sayed AM, Scarborough P, Galea S (2012). Unevenly distributed: a systematic review of the health literature about socioeconomic inequalities in adult obesity in the United Kingdom. BMC Public Health.

[41] Tamayo T, Christian H, Rathmann W (2010). Impact of early psychosocial factors (childhood socioeconomic factors and adversities) on future risk of type 2 diabetes, metabolic disturbances and obesity: a systematic review. BMC Public Health.

[42] Searching for healthy food: the food landscape in California cities and counties [http://www.publichealthadvocacy.org/searchingforhealthyfood.html].

[43] Children’s food environment state indictor report [http://www.cdc.gov/obesity/downloads/ChildrensFoodEnvironment.pdf]

[44] American Diabetes Association (2013). Economic Costs of Diabetes in the U.S. in 2012. Diabetes Care.

